# Lunatic Literature

**Published:** 1887-09-03

**Authors:** 


### LUNATIC LITERATURE.

By a Voluntary Commissioner.

Life in a lunatic asylum must always be more or less of a nursery existence. The chief effort of those in authority must be to keep their grown-up children "good" by every device that can be invented for their amusement; but it does not follow that the games the patients play at are wholly trivial or silly. Many forms of mania, which unfit a man for taking an active part in life, leave his general intellectual and social qualities almost unimpaired, and the exercise of these faculties, besides keeping the patient from becoming morbid, gives to the little world of the asylum its microcosmic labours and ambitions, amusements and castes.

Some of the larger asylums publish a magazine, the contributors to which are patients, and the poems and articles in these journals are by no means so insane as the outside world might expect. Many of the papers deal with the great excitements of the asylum. Lectures, concerts, and balls are reported with a fulness of detail that show how much the writers enjoyed their occurrence. In the description of a dramatic recital, which we take from the pages of *The Morningside Mirror*, the journal of the Edinburgh asylum, even the dresses of the lady performers are described with artistic fervour:—"The three Graces were all dressed alike, in pretty and quaint costumes of green plush or velveteen, the play of light from bright to dark green giving all the appearance of a rich shot-silk velvet (if such there be)."

Less æsthetic sentiments fill the mind of the writer who describes the Christmas, Hallowe'en, and other balls; for besides speaking in favourable terms of the music and the floor, and the vigour with which dancing was kept up, he invariably details what manner of good things were provided for supper. Other paragraphs contain reports of chess contests and cricket matches, with elaborate criticism of moves and runs. In one number is given a metrical description of a game at chess, some of the stanzas of which take a satiric turn:—

"Set all the foot-soldiers
In front of the play,
They've *pawned* their small bodies
For rations and pay;
They're sure to be killed
Ere the nobles appear,
For the latter are prudent
And keep in the rear."

Some of the prose articles, however, are more serious in import than this reporting work. One writer discourses with varied, if not profound, learning on the Odes of Anacreon. That these notes on the Greek poet, in which the author assumes that his readers are familiar with the original Greek, though he occasionally is generous enough to quote from Moore's translation, would meet with popular favour is somewhat doubtful. Probably no one else profited so much by the writing of them as the author himself. More likely to interest the average mind are the reminiscences of "College Life Forty Years Ago,"—though to an outsider there is something pathetic in the inmate of an asylum sitting down to record the habits of days when the life, now so sadly clouded, was illuminated with the hope and energy of youth.

But it is when the patients quit the dull level of prose and, like Mr. Silas Wegg, "drop into verse," that their work shows most originality and freedom. The *Morningside Mirror*, like more famous journals, has its Jubilee Ode, as loyal as need be, though dwelling more on national gains and glories than the effusions of Lords Tennyson and Rosslyn. After describing, with perhaps too much detail, the wondrous works of steam and electricity, and the results of war and colonisation, the poet bursts out in a strain of true John Bull exultation :—

"Who are first in thought and action,
Who are foremost in the strife ?
We. the children of Victoria,
Mother of the nation's life."

Immediately after he falls into a sadder and more practical strain. He anathematises over-education, and gives the advice :—

"Let us not have too much schooling,
Making boys grew nervous, vain ;
Too much brain is worst of fooling,
Let us use our *hands* again."

Returning at the end of his poem to the ostensible subject of it, he addresses to the Queen at least one not unworthy stanza :—

"Sorrow's shafts have often struck thee—
All who live feel grief and wrong—
But the soul is cleared by sorrow,
And the struggle maketh strong."

It is, however, in verse of less serious import that the poet-patients show to most advantage. Though we on the outside of the asylum walls are apt to think that humour is in a very special way a characteristic of sanity, we are forced to admit that a pretty keen sense of the comic possibilities of life and literature is displayed within them. There is a pretty fair mock Spenserian, or at least Shenstonian flavour in the "Elegy on the Lamented Decease of Canis Tauserius," as one stanza may show :—

"At a cat near top of tree high-perched, sublime,
He would loudly bark with glee most strenuously,
And at the foot would wait great length of time,
Barking for hours in wonder's ecstasy ;
And shaking tail, sit 'neath the o'erbranching bower,
To make the woods with doggish tones resound,—
Ceaseless denouncing cats with all his power,
To wake the echoes in the glooms profound !
His fearful barks the neighbours would alarm,
As if a foreign foe had seized the land,
And there were cause to dread some deadly harm,
And he were ambitious of a war-command !"

In the satiric, lightly reflective strain of the *vers de société* is the song, somewhat over-burdened with technical phrases, entitled "The Bard in the Billiard Room"—

"Chalk the cue and pot the red—
You must take it from the D—
Cunning hand and canny head,
What are all your skill to me ?
I have marked your breaks and misses,
Perched aloft above the fray,—
Pockets, cannons, flukes, and kisses ;
Best of all is *one away*.

"Time and change, and wear and tear,
Try all things beneath the blue,
Tunnel through the chalk four-square,
Loose the leather from the cue.
Longest breaks at last are ended ;
Every dog must have its day :
Clouds shall pass, and cues be mended,
And the Bard be *one away*."

Perhaps one of the cleverest things that asylum literature has produced is an ode addressed to the Cheshire cat, a mystic animal known to the readers of *Alice in Wonderland*, which parodies the somewhat verbose measures of Mr. Swinburne's verse :—

"Green eye-balls, that gleam like a station,
When the signals are lit for the night ;
Fierce whiskers and wild exultation
Of a grin full of feline delight :
Swift questions and affable sallies,
Dark answers that never make plain,
And a form that fades wholly, and rallies
To vanish again.

"Nine lives have thy brethren and sisters,
But thy lives will be ninety times nine,
For thy genius is true gold that glisters,
And thy rhymes shall be deathless as mine.
The right of thy race has grown hoary
On the symbols of kingship to gaze ;
Thou hast *grinned* at the crown and its glory,
And then gone thy ways.

"O creature untamed, yet domestic !
O name that makes cheeses taste well !
O mirthfully mouthed and majestic,
Dost thou lodge in a dream or a dell ?
O purring and ponderous presence,
O bright brindled birth of the years !
O pleasantest face in a pleasance,
Untarnished by tears.

"Dost thou tell us, secure in thy greenness,
High up on thy free forest shelf,
'Take care of the sound, of its keenness,
And the sense will take care of itself' ?
That the bell must be more than the chiming,
That the word must be more than the thought,
And that reason must always be rhyming,—
At least that it ought.

"We shall learn how the baker counts dozens ;
We shall learn whether day is not night ;
And our sisters, our aunts, and our cousins,
Why they like to be married in white.
We shall learn if twice eight can make twenty ;
We shall learn if the Thames will ignite,
And of all things conceivable, plenty,—
And perfectly right."

Such examples as these of lunatic literature show the bright side of asylum life, and prove that energy and happiness are not wholly lost by those unfortunates to whom we so often give a half-contemptuous pity. But at best it can be only the silver lining to a most gloomy cloud, "bright by excess of dark." We have spoken of it as a nursery existence. Recalling how many hopes are blighted, ambitions quenched, and hearts wounded by the shadow on the minds of those whose best life-work consists of such trifles as we have quoted, we are tempted to murmur sadly the words of Wendell Holmes :—

"Our nursery joys
We know must have an end,
But love and friendship's broken toys
May God's good angels mend !"

